# Validation of the Italian version of the Cluster Headache Impact Questionnaire (CHIQ)

**DOI:** 10.1007/s10072-023-06758-0

**Published:** 2023-03-20

**Authors:** Agnese Onofri, Luigi Francesco Iannone, Antonio Granato, Gabriele Garascia, Luca Bartole, Paolo Manganotti, Catello Vollono, Marina Romozzi, Costanza Sottani, Paolo Calabresi, Cristina Tassorelli, Grazia Sances, Marta Allena, Roberto De Icco, Francesco De Cesaris, Andrea Burgalassi, Alberto Chiarugi, Carlo Baraldi, Simona Guerzoni, Maria Pia Prudenzano, Adriana Fallacara, Maria Albanese, Innocenzo Rainero, Gianluca Coppola, Alfonsina Casalena, Edoardo Mampreso, Francesca Pistoia, Paola Sarchielli, Marisa Morson, Simona Sacco, Pierangelo Geppetti, Raffaele Ornello, on behalf of the Italian Headache Registry (RICe) Study Group

**Affiliations:** 1 Department of Biotechnological and Applied Clinical Sciences, University of L’Aquila, Via Vetoio 1 Coppito, 67100 L’Aquila, Italy; 2 Section of Clinical Pharmacology and Oncology, Department of Health Sciences, University of Florence, Florence, Italy; 3 Headache Centre, Clinical Unit of Neurology, School of Neurology, Department of Medicine, Surgery and Health Sciences, University Hospital and Health Services of Trieste, ASUGI, University of Trieste, Trieste, Italy; 4 Fondazione Policlinico Universitario Agostino Gemelli IRCCS, Università Cattolica del Sacro Cuore, Rome, Italy; 5 Department of Brain and Behavioral Sciences, University of Pavia, Pavia, Italy; 6 Headache Science & Neurorehabilitation Center, IRCCS Mondino Foundation, Pavia, Italy; 7 Department of Specialist Medicines, Digital and Predictive Medicine, Pharmacology and Clinical Metabolic Toxicology-Headache Center and Drug Abuse, Laboratory of Clinical Pharmacology and Pharmacogenomics, AOU Policlinico di Modena, Modena, Italy; 8 Headache Center, Department of Basic Medical Sciences, Neurosciences and Sense Organs, University of Bari, Bari, Italy; 9 Regional Referral Headache Center, Neurology Unit, University Hospital Tor Vergata, 00133 Rome, Italy; 10 Department of Systems Medicine, University of Rome Tor Vergata, 00133 Rome, Italy; 11 Headache Center, Department of Neuroscience “Rita Levi Montalcini”, University of Torino, Turin, Italy; 12 Department of Medico-Surgical Sciences and Biotechnologies, Sapienza University of Rome Polo Pontino, Latina, Italy; 13 Neurology Unit, “G. Mazzini” Hospital, Teramo, Italy; 14 Headache Centre, Neurology - Euganea, Health Unit, Padua, Padua Italy; 15 Section of Neurology, Department of Medicine and Surgery, University of Perugia, Perugia, Italy; 16 Italian Society for the Study of Headache (SISC), Rome, Italy

**Keywords:** Cluster headache, Questionnaire, Cluster Headache Impact Questionnaire, Quality of life, Disability

## Abstract

**Background:**

The Cluster Headache Impact Questionnaire (CHIQ) is a specific and easy-to-use questionnaire to assess the current impact of cluster headache (CH). The aim of this study was to validate the Italian version of the CHIQ.

**Methods:**

We included patients diagnosed with episodic CH (eCH) or chronic CH (cCH) according to the ICHD-3 criteria and included in the “Italian Headache Registry” (RICe). The questionnaire was administered to patients through an electronic form in two sessions: at first visit for validation, and after 7 days for test-retest reliability. For internal consistency, Cronbach’s alpha was calculated. Convergent validity of the CHIQ with CH features and the results of questionnaires assessing anxiety, depression, stress, and quality of life was evaluated using Spearman’s correlation coefficient.

**Results:**

We included 181 patients subdivided in 96 patients with active eCH, 14 with cCH, and 71 with eCH in remission. The 110 patients with either active eCH or cCH were included in the validation cohort; only 24 patients with CH were characterized by a stable attack frequency after 7 days, and were included in the test-retest cohort. Internal consistency of the CHIQ was good with a Cronbach alpha value of 0.891. The CHIQ score showed a significant positive correlation with anxiety, depression, and stress scores, while showing a significant negative correlation with quality-of-life scale scores.

**Conclusion:**

Our data show the validity of the Italian version of the CHIQ, which represents a suitable tool for evaluating the social and psychological impact of CH in clinical practice and research.

**Supplementary Information:**

The online version contains supplementary material available at 10.1007/s10072-023-06758-0.

## Background

Cluster headache (CH) is a primary headache disorder that affects approximately 0.1% of the population [1]. Approximately 85% of individuals afflicted by CH have episodic CH (eCH), defined by the occurrence of remissions lasting at least 3 months between active bouts, while 15% of patients have chronic CH (cCH), characterized by the lack of remissions lasting more than 3 months [2].

Although less frequent than other headache disorders, CH might be associated with a substantial degree of disability due to the recurrent attacks of excruciating pain. The impact of CH on patients’ life can be severe; it is estimated that almost 20% of patients with CH have lost a job due to their headache, while another 8% are out of work or on disability [3]. CH is also frequently associated with comorbidities, mostly psychiatric, such as depression, anxiety, and aggressive behavior [4, 5].

Patient-reported outcomes (PROs) are important tools to assess the impact of headache disorders on the affected individuals. Contrariwise to migraine, in which the importance of PROs has been widely recognized [6], there is still little attention to the PROs for patients with CH. The available CH-specific questionnaires [7, 8] are rather time-consuming and poorly usable in the everyday clinical practice. More in detail, the Cluster Headache Quality of Life Scale is a 28-item scale focusing on quality of life [7], while the Cluster Headache Scales are a 36-item tool assessing psychosocial factors [8]; notably, those scales do not focus on the disability related to headache itself. The Cluster Headache Impact Questionnaire (CHIQ) is a short questionnaire recently developed in the German language and published along with an English translation [9]. The CHIQ consists of eight items rated on a 6-point Likert scale. Each question receives a score from 0 (“Never”) to 5 (“Always”); the total score is calculated by summing up the scores of each question, for a total range from 0 to 40. Questions refer to the last week and assess limitations in physical and mental daily activities, self-injurious behavior, and the impression of being a burden to the patient’s social environment; additional questions assess attack frequency and acute medication intake during the last week [9]. The original version of CHIQ showed good internal consistency and test-retest reliability, and positive correlations with attack frequency and acute medication intake; positive correlations were also found between the CHIQ score and depression, anxiety, and stress scores, while a negative correlation was found with a measure of quality of life [9], demonstrating the ability of CHIQ to perform a thorough clinical assessment of patients with CH.

The aim of this study was to validate the Italian version of the CHIQ to provide a reliable and easy-to-use PRO for patients with CH in clinical practice and research.

## Methods

### Questionnaire translation

**Fig. 1:**
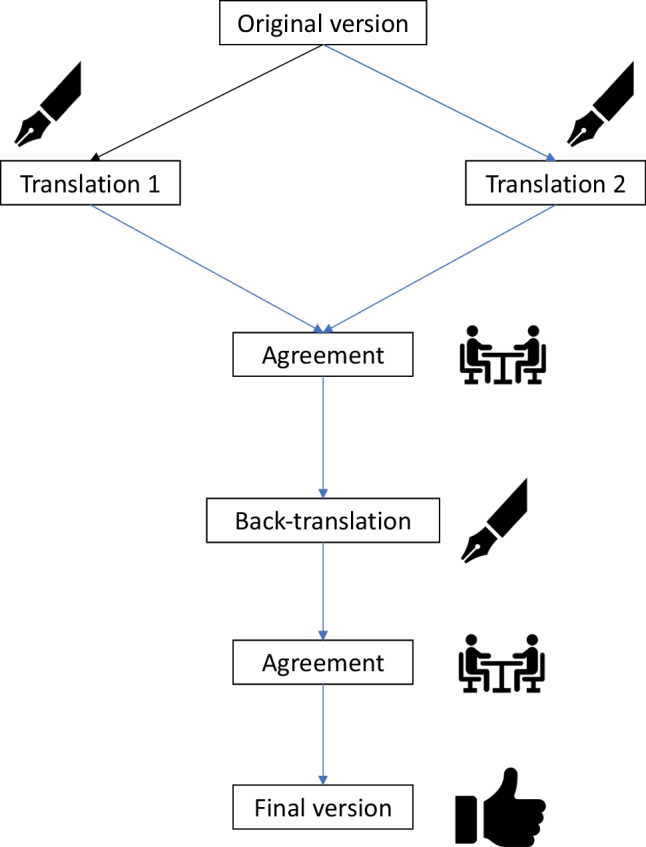
Translation process

We followed international recommendations for the translation protocol [10] (Fig. 1). The English version of the CHIQ questionnaire was first translated from English to Italian by two independent researchers (AO and RO) and then back translated by a native English speaker (MM) to resolve potential discrepancies between the Italian and English version. Conflicts were resolved by discussion and agreement among all the involved researchers. The final questionnaire is provided as supplementary material.

### Study design and selection of the validation cohort

To test the applicability of the questionnaire, patients ≥ 18 years old who met ICHD-3 criteria for eCH or cCH [2] and included in the “*Italian Headache Registry*” (RICe) [11] were recruited between April and December 2022. RICe was approved by the Ethics Committee of the University of Florence (code 14591_oss) and mutually recognized by the other local Institutional Review Boards. The study was conducted according to the guidelines of the Declaration of Helsinki and all patients signed an informed consent before participating in the study.

We included patients seeking care at the 14 headache centers for the first time (1st visit) or for follow-up visit. After obtaining informed consent, patients were asked to complete an online survey conducted using the Research Electronic Data Capture (REDCap) [12] platform combined within the RICe electronic data capture platform. Participants were assigned a personal code in order to match a first and a follow-up survey. The first survey comprised the CHIQ, and the questionnaires to assess psychological dimensions (Depression Anxiety Stress Scale Short Version, DASS-21) [13] and quality of life (Short Form Health Survey, SF-36) [14]. Seven days after the first survey, patients were asked to complete the follow-up survey which included the same questionnaires. Patients with missing data in the CHIQ, DASS-21, or SF-36 questionnaires were excluded.

The validation cohort included patients with active eCH or cCH, while patients with eCH in remission were excluded. Nevertheless, all patients completed the baseline questionnaire; the CHIQ scores of patients with active eCH, cCH, and CH in remission were compared to assess whether the score was able to identify differences across the three groups. Only patients with a stable attack frequency during the week before the baseline and the follow-up assessments (± 2 attacks) were included in the test-retest cohort, similarly to the original CHIQ validation [9].

### Statistical analysis

Demographics and CH characteristics are presented as descriptive statistics (mean ± SD, median [interquartile range, IQR] or numbers, and percentages of patients) if not otherwise specified. Between-group comparisons were performed by non-parametric tests (Wilcoxon or Kruskal-Wallis test, as appropriate) given the assumption of non-normal distribution of variables. For internal consistency, Cronbach’s alpha was calculated. A Cronbach alpha > 0.80 was accepted as good [15]. Test-retest reliability was assessed using intraclass correlation coefficients (ICC). Statistical analysis was performed using R, version 4.2.2 (The R Project for Statistical Computing, Vienna, Austria). Significance was accepted at *p* < 0.05.

## Results

**Fig. 2:**
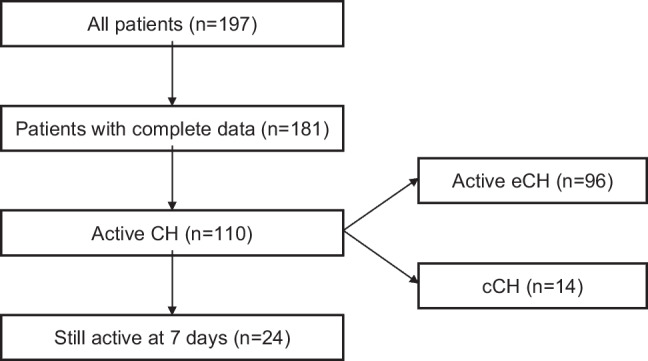
Flowchart of patient selection

During the study period, we evaluated 181 patients with CH of whom 96 (53.0%) had active eCH, 14 (7.7%) cCH, and 71 (39.3%) eCH in remission. The 110 patients with either active eCH or cCH were included in the validation cohort. After 7 days from the baseline questionnaire, 24 (21.8%) of those 110 patients had an attack frequency similar to baseline and were therefore included in the test-retest cohort (Fig. 2).

The characteristics of patients with active eCH, cCH, and eCH in remission are reported in Table 1. The 110 patients of the validation cohort had a median number of 8 headache attacks (IQR 3–15) and used a median of 7 analgesics (IQR 2–12) during the week preceding questionnaire assessments.

**Table 1:** Characteristics of patients included in the study. The validation cohort only included patients with chronic and active episodic cluster headache

Characteristic (*N* = 181)	cCH (*n* = 14)	Active eCH (*n* = 96)	eCH in remission (*n* = 71)	*p* value
Male, *n* (%)	13 (92.9)	77 (80.2)	63 (88.7)	0.057
Age, median (IQR)	56 (46–59)	46 (39–53)	45 (36–52)	0.060
Age at onset, median (IQR)	26 (18–34)	27 (19–37)	24 (18–33)	0.360
Attacks in last week, median (IQR)	7 (5–9)	8 (3–15)	-	-
Acute medication uses in last week, median (IQR)	7 (4–9)	7 (2–13)	-	-
Current preventive medication, *n* (%)	13 (92.9)	53 (55.2)	17 (23.9)	<0.001
Current smoking, *n* (%)	11 (78.6)	57 (59.4)	40 (56.3)	0.300
Current alcohol consumption, *n* (%)	1 (7.1)	16 (16.7)	14 (19.7)	0.513

**Fig. 3:**
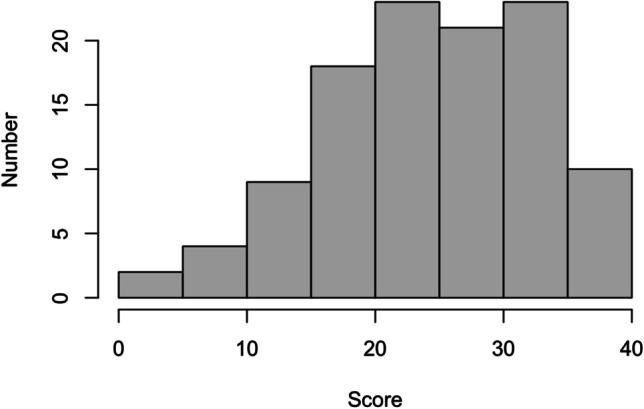
Histogram of Cluster Headache Impact Questionnaire score distribution

In the validation cohort (*n* = 110), the mean (±SD) global CHIQ score was 24.8 ± 8.3 (range 1–39, possible range 0–40). Visual inspection of the CHIQ score histogram revealed a slight positive skewness (Fig. 3). The Shapiro-Wilk test revealed a slight deviation from normality (*W* = 0.976; *p* = 0.044). Internal consistency of the CHIQ was good with Cronbach’s alpha value of 0.891. Cronbach’s alpha did not change after deletion of any of the items (Table 2).

**Table 2:** Results of questionnaire items and Cronbach alpha scores after elimination of each item. Scores are those of patients with active cluster headache (*n* = 110)

Item	Mean (SD)	Cronbach alpha (95% CI)
CHIQ1	3.7 (1.2)	0.870 (0.816–0.906)
CHIQ2	3.6 (1.1)	0.871 (0.821–0.905)
CHIQ3	3.4 (1.3)	0.876 (0.831–0.908)
CHIQ4	3.6 (1.4)	0.870 (0.821–0.903)
CHIQ5	3.4 (1.4)	0.877 (0.828–0.910)
CHIQ6	3.2 (1.4)	0.869 (0.819–0.904)
CHIQ7	1.6 (1.5)	0.904 (0.864–0.931)
CHIQ8	2.4 (1.6)	0.879 (0.830–0.910)
CHIQ score	24.8 (8.3)	0.891 (0.850–0.920)

CHIQ score showed a significant positive correlation with anxiety, depression, and stress scores of the DASS-21 scale, while showing a significant negative correlation with quality-of-life domains assessed by the SF36 scale (Table 3). CHIQ score showed a marginally significant correlation with the number of CH attacks during the previous week, but no significant correlation with the frequency of intake of acute medications (Table 3).

**Table 3:** Correlation between the Italian version of the Cluster Headache Impact Questionnaire and measures of disability, depression, anxiety, stress, and quality of life

Correlation with CHIQ	Spearman’s rho (*p* value)
Attacks in the last week	0.191 (0.046)
Acute medication frequency in the last week	0.145 (0.131)
DASS-D (depression)	0.689 (< 0.001)
DASS-A (anxiety)	0.508 (< 0.001)
DASS-S (stress)	0.722 (< 0.001)
SF-36 (physical functioning)	− 0.282 (0.003)
SF-36 (physical health)	− 0.341 (< 0.001)
SF-36 (emotional problems)	− 0.343 (< 0.001)
SF-36 (energy)	− 0.466 (< 0.001)
SF-36 (emotional well-being)	− 0.587 (< 0.001)
SF-36 (social functioning)	− 0.450 (< 0.001)
SF-36 (pain)	− 0.487 (< 0.001)
SF-36 (general health)	− 0.260 (0.006)

**Fig. 4:**
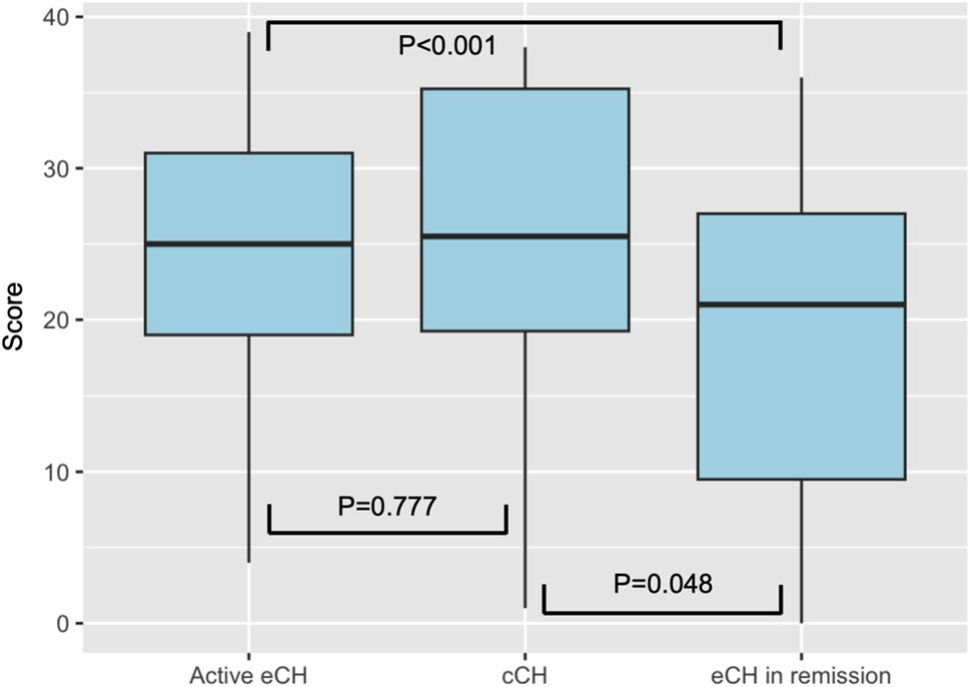
Comparison of Cluster Headache Impact Questionnaire scores across groups

Median CHIQ score was 25 (IQR 19–31) in patients with active eCH, 26 (IQR 19–35) in those with cCH, and 21 (IQR 10–27) in those with eCH in remission. The difference across groups was significant (*p* < 0.001, Kruskal-Wallis test). When comparing groups in couples, the score was similar when comparing patients with active eCH and cCH (*p* = 0.777; Fig. 4), while it was significantly lower in eCH in remission when compared to either active eCH or cCH.

In the test-retest cohort, median weekly headache attacks remained unchanged from 5 (IQR 4–13) to 5 (IQR 2–9) (*p* = 0.284) as were the median CHIQ scores: 25 at baseline (IQR 22–33), 23 at the retest session (IQR 11–30) (*p* = 0.095). The intraclass correlation coefficients were overall poor and ranged from 0.326 and 0.690 (Table 4). Not all coefficients were significant, but the overall score showed a statistically significant test-retest correlation (Table 4).

**Table 4:** Intraclass correlation coefficients for test-retest reliability for patients with active cluster headache 7 days after the first questionnaire (*n* = 24)

Item	Coefficient	95% confidence interval	*p* value
CHIQ1	0.690	0.232–0.882	0.003
CHIQ2	0.417	− 0.095 to 0.753	0.066
CHIQ3	0.532	0.126–0.791	0.006
CHIQ4	0.485	− 0.064 to 0.793	0.045
CHIQ5	0.536	0.131–0.793	0.006
CHIQ6	0.449	0.028–0.745	0.019
CHIQ7	0.326	− 0.165 to 0.683	0.091
CHIQ8	0.594	0.194–0.825	0.003
CHIQ score	0.584	0.156–0.824	0.005

## Discussion

We validated the Italian version of the CHIQ in a nationwide collaboration among 14 headache centers. Our results show good internal consistency, comparable to the original version. Therefore, this version of the questionnaire could be used in clinical and in research settings to evaluate the impact of CH on patients. The availability of a specific PRO for CH will provide clinicians and researchers with an easy-to-use tool that assesses the impact of CH by including additional components beyond the simple quantification of attack frequency and medication use.

Some differences between our validation cohort and the original German cohort are worth mentioning. The first main difference is that we validated our questionnaire in a cohort of patients with CH assessed in headache centers only, while the original CHIQ score was validated in a cohort comprising patients’ associations. Therefore, our cohort might have a higher diagnostic accuracy, as all patients received a diagnosis validated by headache specialists. On the other hand, we reached a lower patient number—mostly referring to patients with cCH—and our results might be less generalizable to the global population of patients with CH. In our cohort, the median number of weekly CH attacks was slightly lower than that of the original validation: 8 headache attacks (IQR 3–15) in our cohort vs 15.2 ± 13.8 attacks in the German cohort. The consumption of analgesics was also lower in our cohort (*n*. 7, IQR 2–12) vs 13.5 ± 14.2 in the German cohort.

The internal consistency of the questionnaire in our cohort was excellent, which makes it suitable for a wider application in Italy. On the contrary, in the test-retest validation, the overall rate of agreement was poor and not significant for some items (Table 3). In our opinion, this result might be due to the low number of patients who still reported headache at the 7-day follow-up. We asked patients to repeat the questionnaire after a shorter time compared with the original validation (7 instead of 14 days). This short timeframe was chosen as we expected that patients referring to headache centers would receive treatments that would quickly change their headache frequency and associated disability. We cannot exclude that effective treatments might have changed headache-related disability even during this short timeframe, thus affecting the reliability of test-retest. This interpretation is supported by the fact that most patients did not report headache at the moment of the follow-up survey.

An additional difference between our cohort and the original CHIQ validation cohort was that we found no difference in median CHIQ scores between patients with active eCH and those with cCH (Fig. 3). This finding is likely influenced by the low number of patients with cCH. The validity of the Italian version of CHIQ as a tool fit for detecting the impact of CH is however strengthened by the significant difference observed between eCH in remission and both groups of active eCH and cCH.

Similar to the original validation, we found a high correlation between CHIQ scores and measures of depression, anxiety, stress, and quality of life. Rather surprisingly, we found only a marginal correlation between the CHIQ scores and CH frequency and no correlation between the scores and acute medication use. This finding might have important implications for the management of patients with CH as it signals that the information coming from the CHIQ questionnaire could provide more complete information compared with those of simple questions about CH frequency or acute medication use.

We performed a validation study on a nationwide cohort of patients referring to headache centers where headache diagnoses are established by trained professionals, therefore increasing the reliability of data. Patient compliance was complete as all patients who were offered the questionnaire on their clinical visit agreed to participate in the study and completed the questionnaire. On the other hand, our study suffers from some potential limitations. We cannot exclude that patients accessing headache centers were more motivated than those attending other care settings. Therefore, a wider application of the CHIQ questionnaire could be associated with some compliance issues compared with the present cohort. Selection bias cannot be excluded as patients were coming from specialized centers. Additionally, the number of patients with cCH and the size of the test-retest cohort were small. The 7-day timeframe for test-retest was also short, which could expose to a risk of recall bias. The validation cohort included 110 patients with a ratio between patients and questionnaire items of 14:1, which is higher than a widely recommended ratio of 10:1 [16], but lower than the optimal 20:1 ratio [17]. For this reason, the reliability of subgroup analyses for eCH and cCH cannot be granted.

## Conclusions

We showed the applicability of the Italian version of the CHIQ in patients with chronic and episodic CH. The questionnaire represents a useful and easy-to-use tool to explore several aspects of disability and quality of life of patients with CH within a limited amount of time. Further large-scale studies are needed to detect relevant cutoffs of the scale associated with clinically relevant differences, to distinguish the disability of patients with eCH and cCH, and to assess the effect of CH treatments on the CHIQ scores as well as on CH frequency and acute medication use.

## Supplementary information

ESM 1

## Data Availability

The raw data collected and analyzed for the current study are available from the corresponding author on reasonable request.

## Abbreviations

CH: Cluster headache
cCH: Chronic cluster headache
eCH: Episodic cluster headache
PRO(s): Patient-reported outcome(s)
CHIQ: Cluster Headache Impact Questionnaire
ICHD-3: International Classification of Headache Disorders, 3rd Edition
RICe: Italian Headache Registry (*Registro Italiano delle Cefalee*)
REDCap: Research Electronic Data Capture
DASS-21: Depression, Anxiety, and Stress Scale Short Version
SF-36: Short Form Health Survey
IQR: Interquartile range
ICC: Intraclass correlation coefficients
