# Supplementary material for: Validation of the Italian version of the Cluster Headache Impact Questionnaire (CHIQ)
Source: Neurol Sci. 2023 Mar 20;44(8):2845–51. doi: 10.1007/s10072-023-06758-0 (PMC10345064; doi:10.1007/s10072-023-06758-0)
Supplement: Supplementary file 1 — ESM 1 [file 10072_2023_6758_MOESM1_ESM.pdf]

Questionario sull'impatto della cefalea a grappolo (Cluster Headache Impact Questionnaire – CHIQ)

Lo scopo di questo questionario è di descrivere l'impatto **ATTUALE** della cefalea a grappolo sulla sua vita quotidiana. **Indichi con una crocetta, per ciascuna domanda, la risposta che meglio descrive la sua condizione nell'ULTIMA SETTIMANA**.

1. Quanto spesso il suo mal di testa ha avuto un impatto sulla sua capacità di lavorare, svolgere le attività domestiche o far fronte ad altre responsabilità?

□ Mai □ Raramente □ Qualche volta □ Spesso □ Molto spesso □ Sempre

2. Quanto spesso il suo mal di testa ha avuto un impatto sulla sua vita familiare, sulle attività del tempo libero o sui contatti sociali?

□ Mai □ Raramente □ Qualche volta □ Spesso □ Molto spesso □ Sempre

3. Quanto spesso si è sentito/a troppo stanco/a per lavorare o svolgere le sue attività quotidiane a causa di mal di testa notturno?

□ Mai □ Raramente □ Qualche volta □ Spesso □ Molto spesso □ Sempre

4. Quanto spesso ha avuto la sensazione di non poterne più o si è sentito/a irritato/a a causa del suo mal di testa?

□ Mai □ Raramente □ Qualche volta □ Spesso □ Molto spesso □ Sempre

5. Quanto spesso ha avuto paura di programmare qualcosa a causa dell'imprevedibilità degli attacchi di mal di testa?

□ Mai □ Raramente □ Qualche volta □ Spesso □ Molto spesso □ Sempre

6. Quanto spesso è stato incapace di pensare lucidamente o di concentrarsi anche tra un attacco e l'altro?

□ Mai □ Raramente □ Qualche volta □ Spesso □ Molto spesso □ Sempre

7. Quanto spesso si è fatto/a del male (ad esempio, battendo la testa o mordendo l'interno della guancia) durante il mal di testa?

□ Mai □ Raramente □ Qualche volta □ Spesso □ Molto spesso □ Sempre

8. Quanto spesso si è sentito/a un peso per gli altri a causa del mal di testa?

□ Mai □ Raramente □ Qualche volta □ Spesso □ Molto spesso □ Sempre

Quanti attacchi ha avuto in totale nell'ultima settimana? _____ attacchi

Quanto spesso ha assunto farmaci per l'attacco acuto di cefalea a grappolo nell'ultima settimana? _____ triptani/ ossigeno
